# Serum levels of CXCL13 are associated with ultrasonographic synovitis and predict power Doppler persistence in early rheumatoid arthritis treated with non-biological disease-modifying anti-rheumatic drugs

**DOI:** 10.1186/ar3742

**Published:** 2012-02-15

**Authors:** Serena Bugatti, Antonio Manzo, Francesca Benaglio, Catherine Klersy, Barbara Vitolo, Monica Todoerti, Garifallia Sakellariou, Carlomaurizio Montecucco, Roberto Caporali

**Affiliations:** 1 Division and Laboratory of Rheumatology, University of Pavia School of Medicine, IRCCS Policlinico San Matteo Foundation, Piazzale Golgi 2, 27100 Pavia, Italy; 2 Biometry and Clinical Epidemiology Service, IRCCS Policlinico San Matteo Foundation, Piazzale Golgi 2, 27100 Pavia, Italy

**Keywords:** Rheumatoid arthritis, CXCL13, synovitis, ultrasonography, biomarkers

## Abstract

**Introduction:**

Biological markers specifically reflecting pathological processes may add value in the assessment of inter-individual variations in the course of rheumatoid arthritis (RA). The current study was undertaken to investigate whether baseline serum levels of the chemokine CXCL13 might predict clinical and ultrasonographic (US) outcomes in patients with recent-onset RA.

**Methods:**

The study included 161 early RA patients (disease duration < 12 months) treated according to a disease activity score (DAS) driven step-up protocol aiming at DAS < 2.4. Clinical disease activity measures were collected at baseline, 2, 4, 6, 9 and 12 months, and US examination of the hands was performed at baseline, 6 and 12 months. Grey-Scale (GS) and Power Doppler (PD) synovitis were scored (0 to 3), with overall scores as the sum of each joint score. CXCL13 levels were measured at baseline by enzyme-linked immunosorbent assay and evaluated in relation to the achievement of low disease activity (LDA, DAS < 2.4) and US residual inflammation (PD ≤ 1) at 12 months.

**Results:**

Baseline levels of CXCL13 were significantly higher in RA compared to healthy controls (*n *= 19) (*P *= 0.03) and correlated with measures of synovitis, such as the swollen joint count (R 0.28, *P *< 0.001), the US-GS (R 0.27, *P *= 0.003) and US-PD (R 0.26, *P *= 0.005) score. Although CXCL13 did not predict the likelihood of achieving clinical LDA at 12 months within a structured treat-to-target protocol, elevated levels of CXCL13 were associated with more frequent increases of methotrexate dosage (*P *< 0.001). Using adjusted analyses, the highest levels of CXCL13 (> 100 pg/ml) were the only independent predictor of residual imaging inflammation (*P *= 0.005), irrespective of initial US-PD scores, disease activity status, acute phase reactants and autoantibodies. Among the patients in clinical LDA at 12 months, US-PD scores ≤ 1 were less frequently achieved in the high baseline CXCL13 (> 100 pg/ml) group, with an adjusted OR = 0.06 (95% CI 0.01 to 0.55, *P *= 0.01).

**Conclusions:**

CXCL13 emerges as a new biological marker in early RA, accurate in assessing the severity of synovitis and the persistence of US-PD activity over time in response to conventional treatments.

## Introduction

Early intervention aiming at suppression of inflammation through intensive steering strategies has remarkably improved outcomes in rheumatoid arthritis (RA) [1,2]. Despite the general effectiveness, response to treatment is highly variable and a substantial number of patients fail to attain adequate clinical targets [3]. Achievement of clinical remission itself may not coincide with true suppression of inflammation, as patients in clinical remission may continue to have synovitis detectable using ultrasound (US) [4]. In this context, the persistence of Power Doppler (PD) activity may predict subsequent joint damage [5] and short-term relapse [6] in disease-modifying anti-rheumatic drug (DMARD)-treated patients in remission.

Prediction of the severity of joint inflammation at an early stage is important to optimise treatment strategies in patients with RA. Unfortunately, individual differences in the course of the disease are not yet accurately explained by traditional factors at baseline, including clinical disease activity, the levels of acute-phase response and autoantibodies [7-9]. Identification of new variables derived from understanding pathogenic mechanisms that correlate more specifically with synovitis, at both the clinical and the sub-clinical level, might refine our ability to build early prognostic algorithms.

The chemokine CXCL13 has features which make it increasingly attractive as a biological marker in RA. CXCL13 is involved in the positioning, cooperation and activation of B and T cells within lymphoid and extra-lymphoid sites [10-12]. In RA synovium, ectopic expression of CXCL13 associates with the local organisation of infiltrating lymphocytes and with the expression of activation-induced cytidine deaminase, an enzyme sufficient and required for somatic hypermutation and class-switch recombination of immunoglobulin genes [13]. Supporting its pathogenic significance, CXCL13 blockade improves disease outcomes in animal models of arthritis [14].

Of relevance to translation from pathobiology to clinical application, synovial CXCL13 mRNA has been recently shown to co-vary with the level of local inflammatory biomarker expression and has been thus proposed as a surrogate marker of synovitis [15]. More importantly, the possibility to detect and quantify the chemokine is not restricted to relatively inaccessible compartments such as the synovial membrane, but also involves the synovial fluid and the peripheral circulation [15,16]. Preliminary results indicate that serum CXCL13 protein levels and synovial CXCL13 mRNA expression significantly correlate, suggesting that the inflamed synovium may constitute an important source of the circulating protein [15]. In line with these data, serum CXCL13 has been proposed as a biochemical marker reflecting the extent of inflammation in a cross-sectional evaluation [17]. The promising value of CXCL13 as a marker in clinical practice has been very recently boosted by a prospective study demonstrating a significant correlation between baseline serum levels and the rate of joint destruction over long-term follow-up [18].

Despite the fact that preliminary data are encouraging, the actual value of CXCL13 in the routine management of RA remains unclear. Indeed, it is currently unknown whether CXCL13 may provide accurate prediction of the burden of the inflammatory process over time. The aim of this study was to investigate whether CXCL13 levels assessed at baseline are associated with disease activity outcomes over a 12-month follow-up in patients with recent-onset RA within a structured treat-to-target protocol. To ensure accurate assessment of joint inflammation, disease activity was measured both clinically and by US.

## Materials and methods

### Study participants and study protocol

Study subjects were 161 recent-onset RA patients recruited from the Early Arthritis Clinic (EAC) of the University Hospital of Pavia based on the availability of baseline serum. The Pavia EAC is a large prospective cohort as previously described [6,19]. All patients followed a disease activity score (DAS)-driven step-up therapeutic protocol aimed at low disease activity (LDA: DAS < 2.4) based on regular clinical assessment (every two months during the first six months, then every three months). Parenteral methotrexate (MTX) was used in monotherapy at the initial dosage of 10 mg/week and progressively increased to 25 mg/week if LDA was not met. Anti-tumour necrosis factor (TNF) therapy was started in case of persistent disease activity above the LDA status despite maximal MTX dosage. Prednisone 12.5 mg/day for two weeks and then 6.25 mg/day was randomly assigned at baseline as part of an open-label therapeutic study on the efficacy of low doses of prednisone in early arthritis involving a larger cohort [19]. At study entry, all patients had a disease duration of less than one year and were DMARD- and glucocorticoid-naïve. The current analysis is based on follow-up data at 12 months, which were available in 155 patients (96%). Baseline characteristics of patients lost at follow-up were not statistically different from those of patients with complete data.

Local Ethical Committee (IRCCS Policlinico San Matteo Foundation) approval was obtained, and all patients signed written informed consent before study entry.

### CXCL13 measurement

Serum samples were collected at baseline (before treatment) and stored at -20°C until analysis. Serum aliquots that had not been thawed before were used.

Measurement for CXCL13 levels was performed in triplicate by colorimetric enzyme-linked immunosorbent assay (ELISA) (R&D Systems, Minneapolis, MN, USA) according to the instructions of the manufacturer. Nineteen healthy subjects were included as controls. Median values of CXCL13 in non-diseased individuals were 53.77 pg/ml (IQR 42.02 to 63.37), comparable to those reported by others [17] and significantly different from median values of RA patients in our cohort (72.74 pg/ml, IQR 46.62 to 116.87, *P *= 0.03).

### Clinical assessment

Patients' characteristics collected at baseline included age, sex, symptom duration, rheumatoid factor (RF) and anti-citrullinated protein antibodies (ACPA) status and titres. Clinical evaluation at each time point (0, 2, 4, 6, 9 and 12 months) included: the number of swollen and tender joints on 44-joint count (SJC, TJC), Ritchie Articular Index (RAI), Global Health assessment (GH) on a 0 to 100 mm Visual Analog Scale (VAS), evaluator global assessment of disease activity (EGA) and patient global assessment of disease activity (PGA) on a 0 to 10 cm VAS. Erythrocyte sedimentation rate (ESR) and C-reactive protein serum levels (CRP) were also measured. At each visit, the disease activity was measured by DAS based on ESR.

Baseline serum levels of CXCL13 were analysed in relation to the proportion of patients achieving LDA according to DAS < 2.4 [20] at the end of follow-up. As a secondary measure, remission rates (DAS < 1.6) were also computed. Clinical outcomes were additionally assessed through the Simplified Disease Activity Index (SDAI) (LDA ≤ 11, remission ≤ 3.3) [21].

### Ultrasonographic assessment

At baseline, 6 and 12 months US was performed by a single experienced operator unaware of clinical data, using a GE Logiq 9 scanner (General Electrics Medical Systems, Milwaukee, WI, USA) with a multi-frequency linear array transducer (10 to 15 MHz), according to the European League Against Rheumatism (EULAR) guidelines [22]. The US assessment included transverse and longitudinal scanning of medial and lateral dorsal view of bilateral wrists (radiocarpal, ulno-carpal, radio-ulnar and midcarpal joints) and metacarpophalangeal joints, as previously described [6]. Synovial PD was evaluated by selecting a region that included bony margins, joint space and a variable view of surrounding tissues. Pulse repetition frequency (PRF) was adjusted at the lowest permissible to maximise sensitivity. Colour gain was set just below the level that causes the appearance of noise artefacts. Flow was demonstrated in two perpendicular planes and confirmed by pulsed wave Doppler spectrum to exclude artefacts. Grey-Scale (GS) and PD signals were assigned to each joint in accordance with semi-quantitative 0 to 3 scales [23]. An overall US score for GS and PD signal was calculated at each US assessment as the sum of either GS or PD signal scores obtained from each joint (range 0 to 36).

Baseline CXCL13 levels were analysed in relation to the proportion of patients achieving a total PD score ≤ 1 at the 12-month assessment, recently tested as a cut-off value for low level imaging activity [24]. As a more stringent criterion, the achievement of a PD score = 0 [24] was also evaluated.

### Statistical analysis

Data were described as mean and standard deviation (SD) or median and interquartile range (IQR) if continuous and as counts and percent if categorical. The prevalence of the considered outcomes was computed together with their exact binomial 95% confidence intervals (95% CI).

The associations of CXCL13 at study entry were assessed with the Spearman R and 95% CI. A multiple linear regression model, with robust standard errors, was fitted to account for potential confounders (age at diagnosis, gender, disease duration and ACPA positivity; none collinear) and partial correlations were computed. Model assumptions were checked by means of residual analysis. Paired comparisons were performed with the paired t test or the Wilcoxon sign rank test.

In outcome analyses, the CXCL13 values were categorised as low or high based on tertile distribution. The association of high baseline CXCL13 (> 100 pg/ml, upper tertile) with clinical and US outcomes at 12 months was evaluated by means of univariable and multivariable logistic models. The goodness of fit test was performed; the c statistic was computed to assess model discrimination. The same confounders as above in addition to glucocorticoid co-medication, the cumulative dose of MTX and other possible predictors of clinical and US disease activity (baseline clinical disease activity and PD scores) were included. Similarly the association of baseline CRP (> 2.3 mg/dl, upper tertile) and ESR (> 39 mm/h, upper tertile) and the US outcome at 12 months was assessed with logistic regression. Finally, the changes over time in the prevalence of the clinical and US endpoints were assessed with a logistic model for repeated measures, while adjusting for baseline disease activity and treatment. For *post-hoc *comparison at each time point the Bonferroni correction was applied. All tests were two-sided and the significance level was set at 5%. Stata 11.2 (StataCorp, College Station, TX, USA) was used for computation.

## Results

### Baseline characteristics of the cohort

Clinical and US characteristics of the population at study entry are summarised in Table 1. The patients had active disease as defined by a DAS ≥ 2.4 (mean 3.61 ± 0.96) and median RA duration of three months (IQR 2 to 6) since their initial clinical symptoms. IgM RF and IgG ACPA positivity was found in 57% and 49% of the patients respectively. At US assessment, GS synovitis was detected in 95% of the patients. A positive PD signal was observed in 76% of the cases, with a median PD score of 3.5 (IQR 1 to 8).

**Table 1:** Demographic, clinical and ultrasonographic data of the study population at baseline

	*N *= 161
Age, years, median (IQR)	64 (50 to 73)
F/M (%)	112/49 (69.6)
Disease duration, months, median (IQR)	3 (2 to 6)
DAS44, mean (± SD)	3.61 (0.96)
SDAI, mean (± SD)	31.53 (13.82)
SJC, median (IQR)	12 (8 to 18)
TJC, median (IQR)	12 (6 to 18)
RAI, median (IQR)	8 (5 to 11)
GH, 0 to 100 mm, median (IQR)	56 (50 to 73)
EGA, 0 to 10 cm, median (IQR)	4.6 (3.5 to 6)
PGA, 0 to 10 cm, median (IQR)	6 (4.8 to 8)
HAQ, median (IQR)	1.25 (0.75 to 1.88)
ESR, mm/h, median (IQR)	28.5 (17 to 46.5)
CRP, mg/dl, median (IQR)	1.19 (0.41 to 3.12)
IgM RF-positive, n. (%)	92 (57.1)
IgM RF titre, UI/ml, median (IQR)	69 (30.5 to 174)
IgG ACPA-positive, n. (%)	79 (49.1)
IgG ACPA titre, UI/ml, median (IQR)	100 (42.7 to 292.5)
US-GS score, median (IQR)	10 (5 to 12)
US-PD positive, n. (%)	122 (75.8)
US-PD score, median (IQR)	3.5 (1 to 8)

ACPA, anti-citrullinated protein antibodies; CRP, C-reactive protein; DAS44, Disease Activity Score in 44 joints; EGA, evaluator global assessment of disease activity; ESR, erythrocyte sedimentation rate; GH, global health assessment; GS, Grey-Scale; HAQ, Health Assessment Questionnaire; Ig, immunoglobulin; IQR, interquartile range; PD, Power Doppler; PGA, patient global assessment of disease activity; RAI, Ritchie Articular Index; RF, rheumatoid factor; SD, standard deviation; SDAI, Simplified Disease Activity Score; SJC, swollen joint count; TJC, tender joint count; US, ultrasonography

### Associations between CXCL13 and disease activity at baseline

In cross-sectional analyses at baseline, serum levels of CXCL13 appeared moderately, although significantly, associated with both CRP (R 0.42, *P *< 0.001) and ESR (R 0.41, *P *< 0.001) levels. Accordingly, CXCL13 behaved as a biomarker of clinical disease activity, measured as either the DAS or the SDAI. Among the core data set measures, CXCL13 was most strongly associated with the SJC (Table 2). The significant association remained when adjustments for possible confounders (age at diagnosis, gender, disease duration and ACPA positivity) were made (Table 2).

**Table 2:** Associations between CXCL13 and clinical and ultrasonographic measures of disease activity at baseline

	Univariable analyses		Multivariable analyses*	
	
	Spearman R (95% CI)	*P*	Partial correlation	*P*
**DAS44**	0.35 (0.21 to 0.48)	< 0.001	0.30	< 0.001
**SDAI**	0.28 (0.12 to 0.43)	< 0.001	0.22	< 0.001
**SJC**	0.28 (0.12 to 0.43)	< 0.001	0.26	< 0.001
**TJC**	0.10 (-0.06 to 0.26)	0.22	-	-
**RAI**	0.12 (-0.04 to 0.28)	0.14	0.06	0.21
**CRP**	0.42 (0.29 to 0.54)	< 0.001	0.38	< 0.001
**ESR**	0.41 (0.27 to 0.53)	< 0.001	0.35	< 0.001
**US-GS score**	0.27 (0.10 to 0.44)	0.003	0.31	< 0.001
**US-PD score**	0.26 (0.08 to 0.42)	0.005	0.32	< 0.001

Results from Spearman R and multivariable linear regression analyses.*Adjusted for age, gender, symptoms duration, ACPA positivity. CRP, C-reactive protein; DAS44, Disease Activity Score in 44 joints; ESR, erythrocyte sedimentation rate; RAI, Ritchie Articular Index; SDAI, Simplified Disease Activity Score; SJC, swollen joint count; TJC, tender joint count; US-GS, Grey-Scale; US-PD, Power Doppler

To confirm the association between CXCL13 and objective measures of joint inflammation, we also examined disease activity assessed by US. A significant correlation was found with both GS and PD scores (Table 2). Again, the significant association remained after accounting for potential confounders (Table 2) as well as for CRP levels (*P *< 0.001, data not shown).

In addition to its specific relationship with clinical and US synovitis, CXCL13 showed further correlation with the autoantibody status. Indeed, CXCL13 levels were significantly higher in ACPA-positive compared to ACPA-negative patients (median values 96.8 pg/ml (IQR 56.4 to 184.2) vs 68.5 pg/ml (IQR 43.5 to 105.9), *P *= 0.001). Differently from CXCL13, neither the ACPA status nor ACPA titres were associated with clinical, laboratory (ESR, CRP) and US measures of inflammation in our cohort (data not shown).

### Clinical and ultrasonographic follow-up of the cohort

By the end of the 12-month follow-up, 67% of the patients were in DAS LDA (62% according to the SDAI) and 41% in remission (11% according to the SDAI). Of the 51 patients (33%) not achieving the target of LDA, 32 had started a combination therapy with anti-TNF according to the treatment protocol. In the remaining 19 patients who did not achieve the target, combination therapy with biological DMARDs was not initiated due to contraindications, denied consent or evidence of disease relapse after reaching LDA at previous visits.

The total PD score significantly fell over the study period, with a mean decrease of 3.23 (95% CI 2.19 to 4.26; *P *< 0.001) at 6 months and 3.78 (95% CI 2.52 to 5.03; *P *< 0.001) at 12 months. In the overall cohort, 78% of the patients showed low level US activity (PD ≤ 1) at the end of follow-up, whilst PD = 0 was observed in 61%. Among the patients achieving a final DAS < 2.4, a PD signal > 1 persisted in 14% of the cases.

### Associations between baseline CXCL13 and clinical disease activity at follow-up

**Figure 1:**
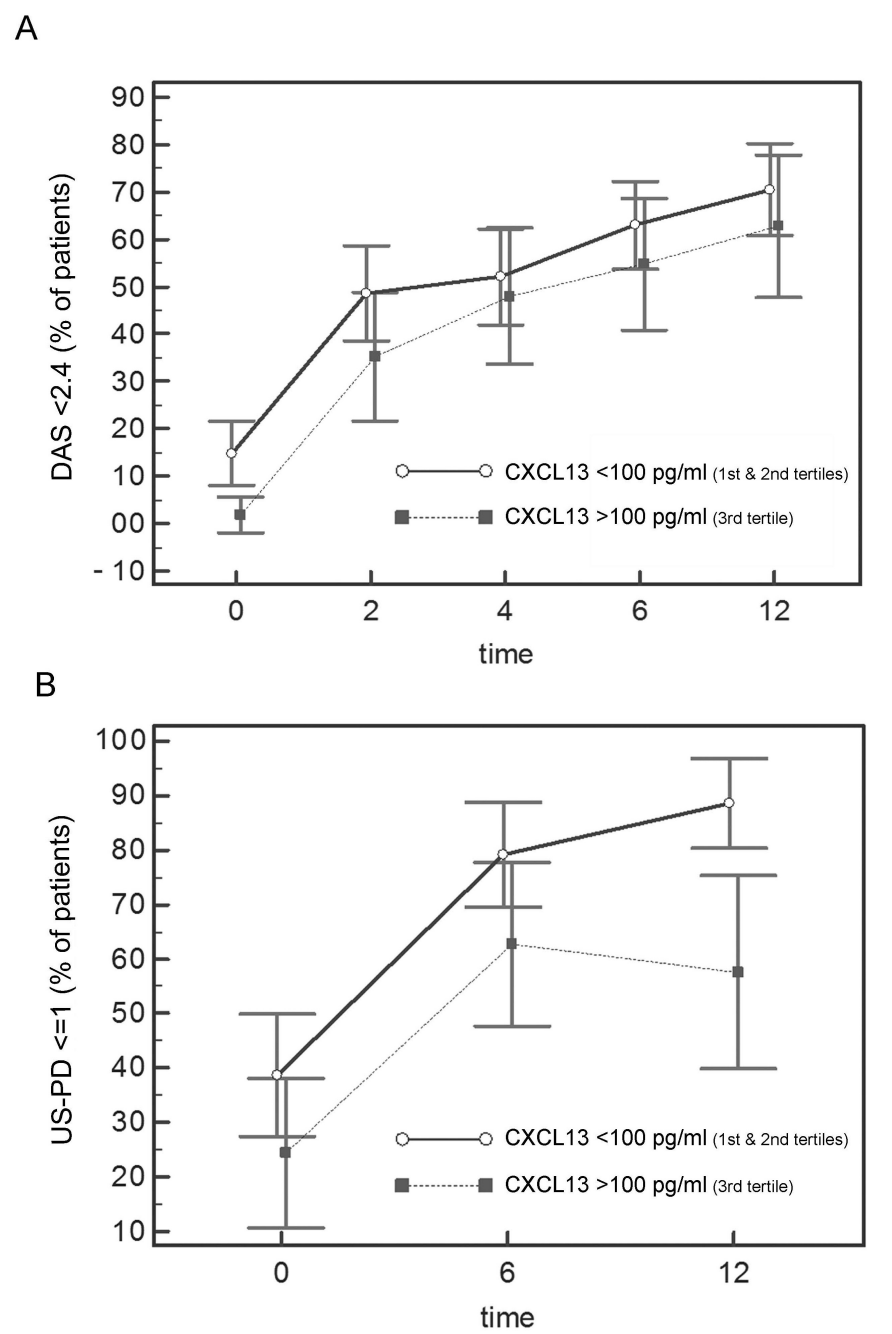
**Clinical and ultrasonographic outcomes over time according to baseline CXCL13 levels**. Baseline CXCL13 levels and progressive achievement of clinical and ultrasonographic (US) outcomes in patients with recent-onset rheumatoid arthritis treated within a structured treat-to-target protocol aiming at low disease activity. Patients were divided into tertiles based on CXCL13 levels. The proportion of patients achieving low disease activity (DAS < 2.4) **(A) **and a power Doppler score ≤ 1 at ultrasonography **(B) **at each time point for different CXCL13 thresholds is shown. The mean ± SD CXCL13 levels were 163.6 ± 63.27 pg/ml for the third tertile (lowest value 99.83 pg/ml), 73.57 ± 12.08 pg/ml for the second tertile and 38.44 ± 10.42 pg/ml for the first tertile. Due to the lack of significant differences between the first and the second CXCL13 tertile, clinical and US outcomes are presented together against the third tertile. Values are the mean ± SD.

Baseline serum levels of CXCL13 were categorised as low or high based on tertile distribution and were studied in association with the rate of achievement of the clinical outcomes at 12 months. Seventy-four patients (71%) with low CXCL13 (first and second tertiles) achieved DAS LDA at 12 months as compared to 30 (59%) with high CXCL13 (third tertile), with an adjusted OR = 1.56 (95% CI 0.62 to 3.94, *P *= 0.35). This lack of association was confirmed when considering as a secondary outcome disease remission, computed through either the DAS or the SDAI (data not shown). As a treatment protocol aimed at LDA may minimise clinical differences due to more frequent therapeutic adjustments in patients not achieving the target, we also examined each time point separately. Overall, the prevalence of LDA increased over time, and was higher in the low with respect to the high CXCL13 group (*P *< 0.001, regression model for repeated measures, adjusted for baseline DAS and treatment), although none of the *post-hoc *comparisons at each time point reached statistical significance (Figure 1A). The median number of therapeutic changes needed by the target protocol was 1(IQR 0 to 2) in the low and 2 (IQR 1 to 2) in the high CXCL13 group (*P *< 0.001) and was independent of baseline DAS and concomitant use of glucococorticoids.

### Associations between baseline CXCL13 and ultrasonographic disease activity at follow-up

The prevalence of PD ≤ 1 at different time points in the low and the high CXCL13 group respectively is shown in Figure 1B (regression model for repeated measures, adjusted for baseline PD and treatment; *P *< 0.001). At 12 months assessment, the proportion of patients achieving low-level imaging activity (PD ≤ 1) was significantly lower in the third CXCL13 tertile (59% vs 88%, *P *= 0.002), with an adjusted OR = 0.16 (95% CI 0.04 to 0.58, *P *= 0.005). The association of high CXCL13 levels and PD persistence was independent of possible confounders (age, gender, disease duration, ACPA positivity, glucocorticoid co-medication and the amount of MTX administered) (Table 3). Notably, CXCL13 levels emerged as the only baseline predictor of US outcomes in our cohort. Indeed, neither CRP nor ESR levels as well as baseline PD scores (all categorised as low or high based on tertile distribution) appeared predictive of the residual imaging activity at 12 months (Table 3). Results were confirmed when considering as an outcome measure the total suppression of PD signal (PD score = 0). The proportion of patients achieving a final PD score = 0 was 70% in the low CXCL13 group compared to 44% in the upper CXCL13 tertile (*P *= 0.02), with an OR = 0.31 (95% CI 0.13 to 0.74, *P *= 0.009). The association became border-line non-significant (*P *= 0.08) after adjusting for pertinent confounders, such as the cumulative treatment and ACPA status (data not shown).

**Table 3:** Predictors of low power Doppler activity (≤ 1) at 12 months

	Univariable analyses		Multivariable analyses*	
	**OR (95%CI)**	***P***	**OR (95% CI)**	***P***

**Overall population**				
CXCL13 third tertile	0.19 (0.07 to 0.54)	0.002	0.16 (0.04 to 0.58)	0.005
CRP third tertile	0.58 (0.21 to 1.56)	0.28	-	-
ESR third tertile	0.61 (0.22 to 1.7)	0.35	-	-
ACPA (positive)	0.40 (0.14 to 1.15)	0.09	0.41 (0.12 to 1.40)	0.15
baseline PD score (third tertile)	0.94 (0.31 to 2.83)	0.91	-	-
**DAS < 2.4**				
CXCL13 third tertile	0.05 (0.01 to 0.42)	0.006	0.06 (0.01 to 0.55)	0.01
CRP third tertile	0.53 (0.12 to 2.37)	0.41	-	-
ESR third tertile	1.22 (0.22 to 6.72)	0.82	-	-
ACPA (positive)	0.46 (0.09 to 2.22)	0.33	-	-
baseline PD score (third tertile)	0.57 (0.11 to 2.86)	0.49	-	-

*Adjusted for age, gender, symptoms duration, glucocorticoid comedication, total amount of methotrexate administered and each of the variables with *P *< 0.2 in univariable analysis.

ACPA, anti-citrullinated protein antibodies; CRP, C-reactive protein; DAS, Disease Activity Score; ESR, erythrocyte sedimentation rate; PD, Power Doppler. CXCL13 third tertile: > 100 pg/ml. CRP third tertile: > 2.3 mg/dl. ESR third tertile: > 39 mm/1 h baseline PD score third tertile: > 7

CXCL13 baseline levels could further discriminate different US outcomes among patients achieving DAS < 2.4 at 12 months. Indeed, the high CXCL13 group less frequently reached low level imaging activity (PD ≤ 1) despite clinical LDA (65% vs 98%, *P *= 0.002), with an adjusted OR = 0.06 (95% CI 0.01 to 0.55, *P *= 0.01). Again, CXCL13 levels appeared the only baseline predictor of PD residual inflammation in patients in clinical LDA (Table 3).

## Discussion

The identification of novel markers measurable in accessible compartments and capable of predicting relevant outcomes in early RA is a critical issue with direct implications in routine clinical practice as well as in enhancing our understanding of the pathobiological processes of the disease. In this study, we provide first time evidence that systemic measurement of the lymphoid chemokine CXCL13 can be exploited to predict synovitis outcomes in patients with recent-onset RA. We demonstrate that, within a structured treat-to-target protocol, baseline serum levels of CXCL13 associate with the severity of synovitis as assessed by US and predict PD persistence after 12 months in response to conventional treatments. The particular setting of an inception cohort of early RA patients (< 12 months from symptom onset), untreated at baseline and homogeneously treated with MTX monotherapy according to a steering strategy enabled us: a) to minimise the influence of possible confounders, such as disease duration and type of medication, on CXCL13 specific associations and predictive values, and b) to determine its cross-sectional and prognostic significance in a "real-life" clinical platform.

In keeping with previous data [17,18], CXCL13 levels at baseline (before treatment) were confirmed to associate with clinical and laboratory measures of disease activity. In particular, CXCL13 appeared as a marker of synovitis, as assessed clinically and, more relevantly, by US. Correlations with imaging synovitis have been also found for ESR and CRP [25,26]. However, an improved face validity of CXCL13 as a marker compared to acute phase proteins is that this chemokine can be induced directly within the rheumatoid joint, where it associates with the development of lymphoid follicles and germinal centre reactions [27,28]. Although serum CXCL13 may originate from a variety of different sources, including lymph nodes where CXCL13 is constitutively produced [11,12] and which can exhibit aspects of reactivity in association with active RA [29], the inflamed synovial tissue appears to significantly contribute to the systemic up-regulation of the chemokine [15]. In contrast, the acute phase proteins are primarily produced by hepatocytes in response to interleukins released during inflammation [30]. Thus, measurement of CXCL13 levels may provide an advantage in the objective assessment of the degree of synovitis compared to routine markers that only indirectly are linked to joint inflammation. As it is now widely accepted that US is more accurate than standard clinical examination at detecting synovitis [25], and PD technologies further improve the capability of identifying actively inflamed joints [31], the association with PD scores found here makes CXCL13 a valuable surrogate marker for quantitative assessment of objective and pathologically relevant disease processes in RA.

The specific association with US parametres might explain why CXCL13 assessment at baseline was still associated with synovitis evolution at 12 months in our cohort. Patients with CXCL13 levels in the highest tertile (> 100 pg/ml), although comparable to patients in lower tertiles in the rate of achievement of clinical LDA in the context of a treat-to-target strategy, required more frequent dose adjustments of MTX and, accordingly, showed delays in the accomplishment of treatment goals. Even more relevantly, the likelihood of suppression of joint inflammation, as assessed by US measurement of PD signal, was reduced for higher CXCL13 baseline levels. The predictive value of CXCL13 appears of utmost clinical importance when considering patients in LDA states, in which different thresholds of residual imaging synovitis might be of importance for subsequent clinical and radiological evolution [4-6]. Notwithstanding the association of CXCL13 with CRP levels as well as the ACPA-status, both regarded as indicators of less favourable outcomes in RA [32,33], PD activity at 12 months in our cohort was uniquely explained by different levels of the chemokine. Thus, from a clinical perspective, the assessment of baseline CXCL13 provides information on a relevant measure of disease severity not yet predicted by routine variables.

Collectively, our results fully fit with recent observations from Meeuwisse *et al. *[18], providing direct explanation of their potential biological basis. In Meeuwisse's study [18], higher CXCL13 levels were reported to be associated with increased rates of joint destruction in the long term. Although radiographic outcomes in our cohort could not be determined, our data complete previous findings demonstrating that CXCL13 is associated with the intensity of active joint inflammation as assessed by PD. The relationship between imaging-detected synovitis and subsequent structural damage is well established. Reports of several longitudinal studies [31,34,35] have described the persistent PD signal in affected joints being a predictor of radiologic progression in RA. The higher rates of PD signal persistence that we report in patients in the third CXCL13 tertile, thus fully fit with increased progression of radiographic damage. Meeuwisse *et al. *[18] also reported that higher CXCL13 levels were predictive of significantly lower chances of achieving drug-free remission during seven years' follow-up. Persistence of a PD signal above low imaging activity in these patients provides a possible explanation, as US signs of ongoing inflammation are associated with unstable remission and short-term disease relapses [6]. Also, later time points at which LDA were attained during the first year of the disease in patients in the third CXCL13 tertile in our cohort further justify lower rates of drug-free remission. Indeed, it is well established that one of the major determinants of good clinical outcomes is the time that elapses to achieve the outcome itself [36,37].

Although the results of this study provide a solid foundation for our conclusions, the study does have some limitations. Definite confirmation of the specific relationship between serum CXCL13 and PD synovitis would require paired longitudinal assessments at each time point during follow-up, a possibility that was limited in our study due to the unavailability of serum samples matched with US at follow-up. Another relevant issue remains the direct demonstration of the histopathological correlates of serum CXCL13 in relation to specific synovial cell populations and inflammatory patterns, which is currently being developed in our laboratory. As synovial CXCL13 protein and mRNA associate with lymphocyte infiltration [38], the assessment of CXCL13 levels in the peripheral circulation might turn out as a comprehensive, non-invasive tool to gain further information on the clinical significance of synovial tissue heterogeneity in RA [39,40].

## Conclusions

The Outcome Measures in Rheumatology (OMERACT) group continues to develop validation criteria for a soluble biomarker which reflects relevant outcomes in RA [41]. Although different sources of variation in serum levels have to be further investigated, CXCL13 is acquiring increasing validity as a new soluble marker in RA according to the OMERACT filter. The relationship between CXCL13 and an objective measure of synovitis, such as US-PD, as well as its independent predictive value for PD persistence within an unselected cohort of patients with recent onset RA that we report in this study add robustness to previous data. Our findings appear further strengthened by the plausible biological explanation that links CXCL13 with synovial pathology. In conclusion, we suggest that CXCL13 is a promising prognostic marker in early RA, accurate in assessing the severity of synovitis and its persistence over time in response to conventional treatments.

## Abbreviations

ACPA: anti-citrullinated protein antibodies; CI: confidence interval; CRP: C-reactive protein; DAS: Disease Activity Score; DMARD: disease-modifying antirheumatic drug; EAC: Early Arthritis Clinic; EGA: evaluator global assessment of disease activity; ESR: erythrocyte sedimentation rate; GH: global health assessment; GS: Grey-Scale; HAQ: Health Assessment Questionnaire; Ig: immunoglobulin; IQR: interquartile range; LDA: low disease activity; MTX: methotrexate; PD: Power Doppler; PGA: patient global assessment of disease activity; PRF: pulse repetition frequency; RA: rheumatoid arthritis; RAI: Ritchie Articular Index; RF: rheumatoid factor; SD: standard deviation; SDAI: Simplified Disease Activity Score; SJC: swollen joint count; TJC: tender joint count; TNF: tumour necrosis factor; US: ultrasonography; VAS: Visual Analog Scale.

## Competing interests

The authors declare that they have no competing interests.

## Authors' contributions

SB and AM were the principal investigators for the study, responsible for the study design and the analysis and interpretation of data as well as for writing the manuscript. FB was responsible for the quantification of serum CXCL13 and gave constructive comments on the manuscript. CK performed the statistical analysis and critically revised the manuscript. BV assisted in the quantification of serum CXCL13 and participated to the interpretation of data. MT participated in clinical assessments and performed all the ultrasonographic examinations. GS performed all the clinical assessments. CM facilitated the performance of the study, giving input to the study design and giving constructive comments on the manuscript. RC participated in the study design, critically revised the manuscript and gave final approval. All the authors have read and approved the final manuscript.
